# A novel scheme for the validation of an automated classification method for epileptic spikes by comparison with multiple observers

**DOI:** 10.1016/j.clinph.2017.04.016

**Published:** 2017-07

**Authors:** Niraj K. Sharma, Carlos Pedreira, Maria Centeno, Umair J. Chaudhary, Tim Wehner, Lucas G.S. França, Tinonkorn Yadee, Teresa Murta, Marco Leite, Sjoerd B. Vos, Sebastien Ourselin, Beate Diehl, Louis Lemieux

**Affiliations:** a Dept. of Clinical and Experimental Epilepsy, UCL Institute of Neurology, London, United Kingdom; b Dept. of Experimental Psychology, University of Oxford, Oxford, United Kingdom; c Translational Imaging Group, Centre for Medical Image Computing, UCL, London, United Kingdom; d Dementia Research Centre, UCL Institute of Neurology, London, United Kingdom

**Keywords:** Interictal spike classification, Intracranial EEG, Automated spike classification, Information theory

## Abstract

**Objective:**

To validate the application of an automated neuronal spike classification algorithm, *Wave_clus* (WC), on interictal epileptiform discharges (IED) obtained from human intracranial EEG (icEEG) data.

**Method:**

Five 10-min segments of icEEG recorded in 5 patients were used. WC and three expert EEG reviewers independently classified one hundred IED events into IED classes or non-IEDs. First, we determined whether WC-human agreement variability falls within inter-reviewer agreement variability by calculating the variation of information for each classifier pair and quantifying the overlap between all WC-reviewer and all reviewer-reviewer pairs. Second, we compared WC and EEG reviewers’ spike identification and individual spike class labels visually and quantitatively.

**Results:**

The overlap between all WC-human pairs and all human pairs was >80% for 3/5 patients and >58% for the other 2 patients demonstrating WC falling within inter-human variation. The average sensitivity of spike marking for WC was 91% and >87% for all three EEG reviewers. Finally, there was a strong visual and quantitative similarity between WC and EEG reviewers.

**Conclusions:**

WC performance is indistinguishable to that of EEG reviewers’ suggesting it could be a valid clinical tool for the assessment of IEDs.

**Significance:**

WC can be used to provide quantitative analysis of epileptic spikes.

## Highlights

• We created a validation method for the evaluation of automated classification of interictal spikes.
• We used a modified version of *Wave_clus* (WC) to automatically classify the data of 5 patients.
• WC classification was similar to EEG reviewers providing an unbiased evaluation of the clinical data.

## 1 Introduction

As part of standard practice for assessing patients with epilepsy, clinical neurophysiologists are able to detect interictal epileptiform discharges (IED or ‘epileptic spikes’) during interictal EEG recordings. Although there is no gold standard as to what constitutes an epileptic spike, they tend to comprise a high amplitude deflection event lasting approximately 40–100 ms (De Curtis and Avanzini, 2001). Some patients evaluated for resective surgical treatment for epilepsy are investigated with intracranial EEG (icEEG) usually when there is strong evidence of an epileptogenic focus but not sufficient information to define a surgically resectable area using non-invasive methods. These patients may be implanted with multiple electrodes targeting deep areas of the brain or placed on the cortex to record epileptic activity (Fernández and Loddenkemper, 2013). In these patients, evidence suggests that a good postsurgical outcome is associated with the removal of the region generating the most frequent epileptic spikes (Asano et al., 2003, Marsh et al., 2010). However, detection of epileptic spikes on icEEG has shown a low level of agreement (<50%) for both the intra-rater (Brown et al., 2007) and the inter-rater comparisons between clinical neurophysiologists (Dümpelmann and Elger, 1999, Barkmeier et al., 2012, Gaspard et al., 2014). To reduce this subjectivity, computational algorithms designed for the automated detection of IEDs on icEEG have been implemented (Dümpelmann and Elger, 1999, Bourien et al., 2005, Valenti et al., 2006, Brown et al., 2007, Barkmeier et al., 2012, Gaspard et al., 2014). However, to our knowledge, the work on IED classification has been limited (Bourien et al., 2005, Yadav et al., 2011, Janca et al., 2013).

Classification of IEDs into various IED ‘populations’ generally relies on clinicians distinguishing between different IED types by assessing the EEG waveform which often takes into account the epileptic spike’s field distribution (Gotman, 1999, James et al., 1999), which may also help highlight the boundaries of the region responsible for generating them (the so-called irritative zone). A previous study by our group (Pedreira et al., 2014) demonstrated the successful use of an automated neuronal spike classification algorithm, *Wave_clus* (WC) (Quian Quiroga et al., 2004), to classify epileptic spikes on scalp EEG for the purpose of modelling the concurrently acquired functional MRI. In this study we present and apply a validation framework for a similar application of WC to icEEG recordings (for the purpose of modelling concurrent fMRI data; which will be the topic of future work).

Our aim was to compare human expert IED classification as it is performed in normal (‘optimal’) conditions against the automated classification method to be used with WC. To our knowledge no formal comparison of automated vs human observer classification of epileptic spikes on icEEG has been published to date. Our approach targets the following questions:

• Does WC-human epileptic spike classification agreement variability fall within inter-human classification agreement variability?
• Looking at the classification labels (or clustering groups) of individual spikes; are WC results similar to those of human observers?

To validate this framework we used data from 5 patients reviewed by 3 human observers for the comparison with WC. We hypothesise that WC can produce similar IED classification results to that of human EEG reviewers whilst also providing additional information.

## 2 Data and methods

### 2.1 Patients, icEEG recording and pre-processing

We analysed icEEG signals recorded in 5 right-handed men (24–39 years) who were undergoing simultaneous intracranial EEG-fMRI (Table 1). The five patients were selected based on the small number of polyspikes observed during the recording. All patients underwent intracranial EEG recordings for clinical purposes to delineate the ictal onset zone and/or to perform direct electrocortical stimulation following a recommendation of a multidisciplinary team meeting. Patients were invited to undergo simultaneous intracranial EEG-fMRI (icEEG-fMRI) recordings at the end of their clinical evaluation. This study was approved by the Joint UCL/UCLH Committees on the Ethics of Human Research, and the patients gave written informed consent. The icEEG recording obtained during the simultaneous icEEG-fMRI study was used since we ultimately want to apply WC in the analysis of icEEG fMRI data however, no fMRI data was analysed for the purpose of this study.

**Table 1:** Patient implantation summary and the channels of interest selected for all patients. R: right, L: left, A: anterior, P: posterior.

Patient	1	2	3	4	5
Type of epilepsy	FLE	FLE	FLE	TLE	TOLE
Implantation summary	L superior (SFG), middle (MFG) and inferior (IFG) frontal gyrus. L precentral gyrus. L central sulcus and part of postcentral sulcus. L superior frontal sulcus. L postcentral regions	L frontal lobe (laterally and inferiorly). L M (MFG) and I (IFG) frontal gyrus. L frontal pole	R A and P insula. R A (R ASMA) and P(R PSMA) supplementary sensorimotor areas. R A, M and P cingulum (P C)	R and L amygdalae (R A). R and L hippocampi	Lateral temporal. Temporooccipital junction
Number of icEEG contacts (+channel label)	One 8 × 8 contact grid (G). Two 4-contact depths (DA & DP) One 2 x 8 contact grid (GA)	One 8 × 8 contact grid (GA). One 2 × 8 grid (GD) Two 6-contact depths (DA & DP). Two 6-contact strips (GC & GB)	Two 6-contact depths (ASMA & PSMA) Three 8-contact depths (AC, MC & PC)	Five 6-contact depths (LA, LAH, LPH, RA & RH)	One 4 × 8 grid (GA) One 4 × 5 grid (GP) Three 6-contact strips (SAT, SMBT & SPBT)
Channels of interest	G4 G5 G13 G20 G21 G22 G23 G29 DP2 DP3	GA50 GA51 GA52 GA53 DA4 DA5	ASMA1 ASMA2 ASMA3 PSMA1 PSMA2 PSMA3 PC4 PC5 AI5 AI6	LAH1 LAH2 LPH1 RA1 RA2 RA3 RH1	GA1 GA2 GA9 GA10 GA11 GA17 GA18 DH1 DH2 SAT3 SAT4 SPBT4 SPBT5 SPBT6

In each patient there were between 31 and 84 implanted electrode contacts on configurations including grid electrodes, depth electrodes or both. The electrodes were connected to an MR-compatible amplifier system (Brain Products, Gilching, Germany). icEEG signals were acquired at a sampling rate of 5 kHz. After recording, we applied offline correction for MR scanning artefacts (Allen et al., 2000) and the resulting EEG was down sampled to 250 Hz. The EEG was band-pass filtered (2–70 Hz) and the same referential montage was used for all 4 EEG reviewers.

### 2.2 IED detection

**Fig. 1:**
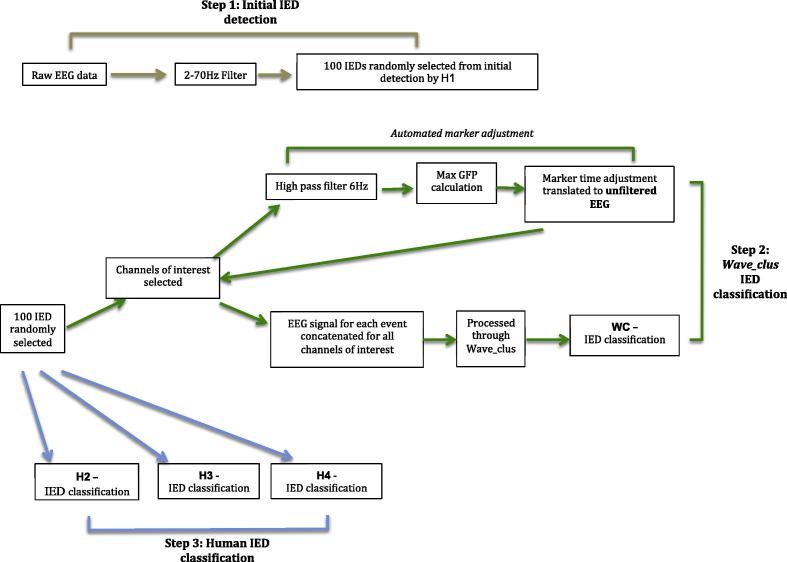
EEG reviewer and WC classification: Step 1: Initial IED detection of 100 lEDs carried out by HI. Step 2: The 100 lEDs detected by HI are classified by *Wave_clus.* This involves selecting channels of interest and adjusting the marker of the sharp wave according to GFP. Step 3: The same set of 100 lEDs detected by HI are independently classified by 3 EEG reviewers H2, H3 and H4. These three steps are carried out for all patients.

The 5 icEEG recordings were inspected by EEG reviewer ‘H1’ for clinical purposes using *BrainVision Analyser* (Brain Products, Germany). During this procedure H1 placed a marker close to the negative/positive peak of each IED event (across the entire recording) that had a single sharp component. We then randomly selected 100 IEDs, using a random number generator, from each recording for this study (see Fig. 1; step 1).

### 2.3 IED classification by human observers (H2, H3 and H4)

Reviewers H2 (10 years of experience in icEEG interpretation), H3 (4 years of experience in icEEG interpretation) and H4 (2 years of experience in icEEG interpretation) independently classified the IED events selected by H1 through visual inspection of the waveforms in a 300 ms time window using BrainVision Analyzer. H2-4 performed the classification by visualizing the EEG activity in all recorded channels, in order to replicate their standard modus operandi. For each patient they were asked to classify the events into IED classes or as non-IEDs. H2-4 were free to define and use as many IED classes as they felt appropriate for each recording. Of the three EEG reviewers, two (H2 and H3) were trained at the same institution. Implantation diagrams, showing the position of the electrodes in relation to the brain, were provided.

### 2.4 Automated IED classification (WC)

The automated classification method *Wave_Clus* is a modification of the one described in Pedreira et al. (2014) and summarised in a flowchart (see Fig. 1; step 2). First, between 8 and 14 channels of interest were selected for each patient based on channels in which the IEDs were noted in the clinical EEG report as being most prominent and frequent. Second, we modified the IEDs’ temporal marking (by H1) by automatically adjusting them to the peak of the sharp wave across the channels of interest (details of this process can be found in Supplementary Methods 1.0).

The IEDs were segmented in 300 ms epochs around the peak of the sharp wave (100 ms pre-peak to 200 ms post-peak) and concatenated across the channels of interest to form meta-IEDs (Pedreira et al., 2014). WC was then used to perform automated classification on the meta-IEDs similarly to our previous work (Pedreira et al., 2014). Based on the morphology and distribution of the IEDs, the algorithm automatically determined the number of classes per case and the events assigned to them. Then, the user performed a visual verification of the final classes obtained; including some events which were labelled as ‘non-IED’.

### 2.5 Automated IED classification validation

We wanted to answer the question: can the results of the automated classification be distinguished from those obtained from humans? More specifically, we compared the two types of IED classification in two ways: first, we determined whether WC-human reviewer agreement variability falls within inter-human reviewer agreement variability; second, we compared *Wave_Clus* and human reviewers’ classifications in terms of comparing IED identification and classification between *Wave_Clus* and all H reviewers.

#### 2.5.1 Does WC-Human IED classification variability fall within inter-human variability?

##### 2.5.1.1 Variation of information (VI)

We compared *Wave_Clus*-human classification agreement variability to inter-human classification variability at a summary level. To this effect we calculated the variation of information (*VI*) between classifications in a pair-wise fashion. The variation of information is a general method to assess the relationship (distance) between two classifications (partitions) of elements (IEDs in this case) (Meilă, 2007). One can quantify the variation of information using the following equation:

(1) $${\mathrm{VI}}_{\text{X,Y}}=-\underset{i\text{,}j}{\sum }r_{\mathrm{ij}}\left[\log2\left(\frac{r_{\mathrm{ij}}}{p_{i}}\right)+\log2\left(\frac{r_{\mathrm{ij}}}{q_{j}}\right)\right]$$

where $p_{i}$ = number of IEDs in class *i* for X, $q_{j}$ = number of IEDs in class *j* for Y, $r_{\mathrm{ij}}$ = number of IEDs classified as *i* by X and *j* by Y. Therefore, for each classifier pair *VI* quantifies how similar the classification results were. Two classifications with perfect agreement have a VI value of 0. In order to determine a threshold of similarity between two classifications, we generated randomised surrogate classifications for 50 artificial observers (see Supplementary Methods 2.0); two classifications were considered similar if their VI value was below the mean of VI minus 2 SD from the surrogate sample.

To compensate for the small sample size, non-parametric bootstrapping (Singh and Xie, 2008) was used on the 100 IEDs for each classification pair. As a result, 1000 *VI* values were calculated for each classifier pair.

To compare the performance between WC and H classifications, the *VI* values for all possible WC-H pairs (WC-H2, WC-H3, WC-H4) were merged to represent *Wave_Clus* classification agreement as a whole (WC_all), and all possible human expert classification agreement H-H pairs (H2-H3, H2-H4, H3-H4) were merged to give an overall human classification agreement (H_all). If *Wave_Clus* is to be applied practically then it is probably preferable that it performs in a way that is indistinguishable from humans, and therefore, WC_all and H_all distribution should overlap. We calculated the Bhattacharyya coefficient (Kailath, 1967, Comaniciu et al., 2000) to measure the percentage of the distribution overlap between WC_all and H_all.

#### 2.5.2 Does *Wave_Clus* produce similar IED marking and classifications to H reviewers?

##### 2.5.2.1 IEDs vs non-IEDs

First, we considered an event labelled as an IED by reviewer H1 to be a “true” IED if at least two of the reviewers, H2-4, labelled it as an IED. If two reviewers of H2-4 labelled an event as a non-IED, we considered it a non-IED for the purpose of this study (Barkmeier et al., 2012, Gaspard et al., 2014). Second, we calculated the sensitivity and the specificity for each classifier (H classifiers and *Wave_Clus*). Then we compared *Wave_Clus* sensitivity and specificity with the ones obtained from the 3 reviewers H2, H3 and H4. We used the pair-wise Cohen’s Kappa statistic to assess the inter-rater agreement for all possible H classifier pairs, with a kappa value >0.4 noted as a high inter-rater agreement (Zijlmans et al., 2008).

##### 2.5.2.2 Visual comparison of IED classes and classification overlap

**Fig. 2:**
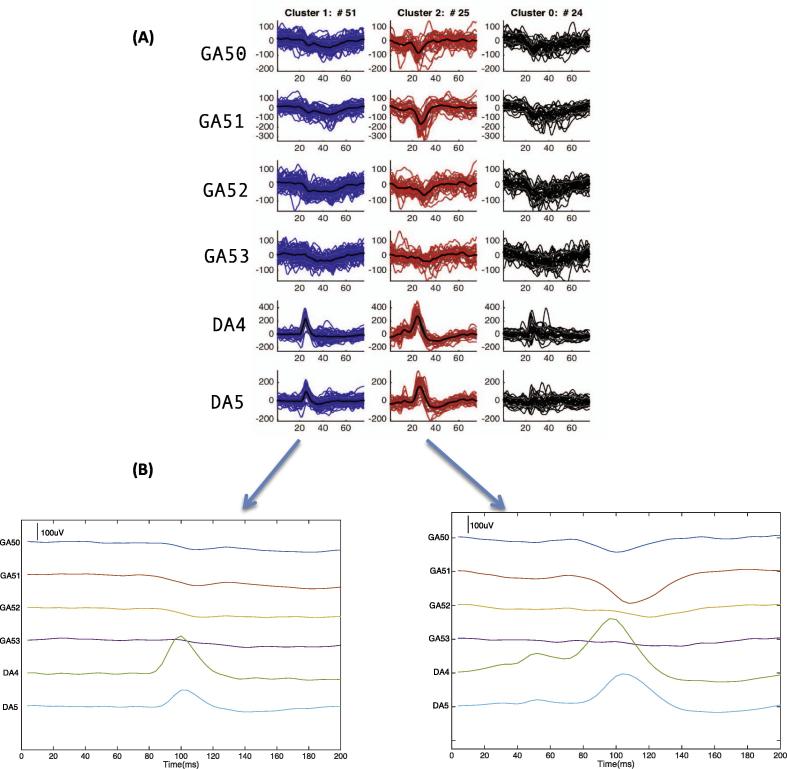
*Wave_clus* clustering results for Patient 2. (A) Output of *Wave_clus* classification. (B) Average waveform of the IED classes over 200 ms.

In order to compare the similarity between WC and H IED classes, the average of the IEDs (over 200 ms) in each WC class was calculated and plotted (see Fig. 2). The average WC class was compared visually to the classes of each EEG reviewer. In addition to this, the agreement *A*_i,j,_ between WC class *i* and H class *j* was calculated as a percentage (the classification overlap):

(2) $$A_{\mathrm{ij}}(\mathrm{WC}\text{;}H)=\left(\frac{r_{\mathrm{ij}}}{\left|{\mathrm{WC}}_{i}\right|}\right)\times 100$$

where |*WC_i_*| is the number of IEDs in WC class *i*, *r_ij_* *=* the proportion of IEDs labelled as WC*_i_* and H*_j_*. The H class with the greatest agreement with each WC class was noted.

## 3 Results

### 3.1 IED classification by human observers (H2, H3 and H4) and WC

The agreement between different classifiers (either H or WC) was not perfect and no two classifications were identical in any given patient. Furthermore, the number of IED classes varied across patients (range: 1–8). Across the group, *Wave_clus* identified 15 classes, 23 classes were identified by H2, 20 classes were identified by H3 and 24 classes were identified by H4 (see Table 2).

**Table 2:** Number of classes assigned by WC, H2, H3 and H4.

EEG classifier	Patient				
	1 (# IED classes + # non-IED)	2 (# IED classes + # non-IED)	3 (# IED classes + # non-IED)	4 (# IED classes + # non-IED)	5 (# IED classes + # non-IED)
WC	3 + 1	2 + 1	2	5 + 1	3
H2	8 + 1	1 + 1	3	6 + 1	5
H3	6 + 1	1 + 1	3	6 + 1	4
H4	6 + 1	4 + 1	3	5 + 1	6

### 3.2 Automated IED classification validation

We present here the results of the analysis for the 3 H observers and WC classifications, following the procedure described in the methods section to address the questions: *Does WC-Human IED classification variability fall within inter-human variability?* And *Does Wave_clus obtain similar IED marking and classifications to H reviewers?*

#### 3.2.1 Does WC-Human IED classification variability fall within inter-human variability?

The mean (SD) for the randomly generated VI values was 408.60(49.29). Looking at the classification agreement at the individual classifier pair-wise level, the overlap values ranged between [239–288] for patient 1, [121–222] for patient 2, [80–135] for patient 3, [169–211] for patient 4 and [77–167] for patient 5 (see Table 3). The *VI* distribution for each classification pair was significantly different from the randomly generated distribution for both H-H pairs and WC-H pairs (*p* < 0.05; see Table 3 for details).

**Table 3:** Variation of information for all classifier pairs and the VI distribution overlap between WC_all and H_all for all patients.

Classification pair	Patient				
	1	2	3	4	5
WC-H2	288.56**	220.21**	112.1**	211.44**	109.28**
WC-H3	239.82**	162.54**	117.49**	169.51**	77.58**
WC-H4	276.13**	206.6**	135.97**	179.87**	146.08**
H2-H3	252.06**	121.74**	83.61**	172.49**	128.2**
H2-H4	262.3**	222.14**	84.2**	188.9**	167.29**
H3-H4	256.17**	134.91**	80.28**	169.75**	129.63**

Overlap (%) (WC_all/H_all)	93.4	66.3	58	96.4	81.1

** Significance at *p* < 0.05.

**Fig. 3:**
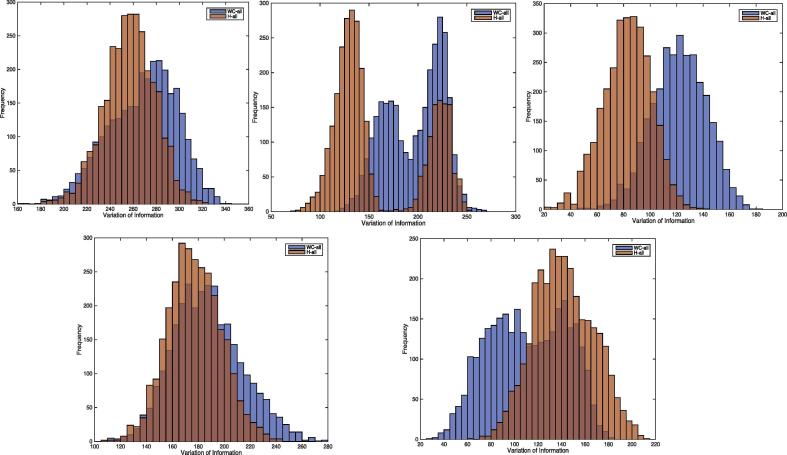
VI distribution for WC_all (blue) and H_all (orange). 1st row (left to right): Patient 1, 2 & 3; 2nd row (left to right): Patient 4 & 5. The values for the null distribution are: mean = 408.60 and SD = 49.29. (For interpretation of the references to color in this figure legend, the reader is referred to the web version of this article.)

Fig. 3 shows the *VI* results for each patient for WC_all and H_all. The *VI* distribution overlap between WC_all and H_all were: 93.4% for patient 1, 66.3% for patient 2, 58% for patient 3, 96.4% for patient 4, 81.1% for patient 5 (see Table 3). Therefore, WC classification falls within inter-human variation.

#### 3.2.2 Does *Wave_clus* obtain similar IED marking and classifications to H reviewers?

##### 3.2.2.1 Sensitivity and specificity: IED vs Non-IEDs

**Fig. 4:**
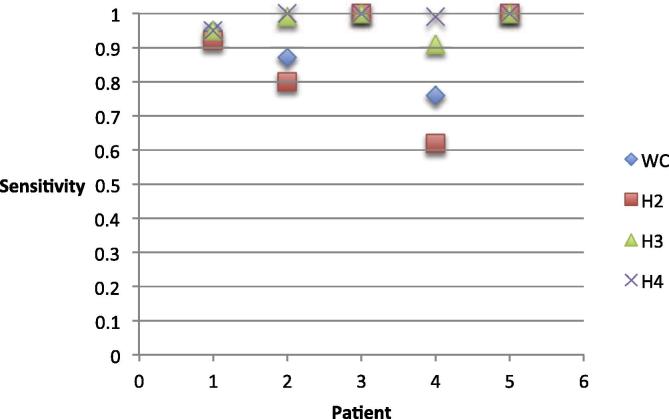
Sensitivity of IED marking of WC and H2, H3 and H4 for all 5 patients.

Across the group, IED detection sensitivity was in the range [0.76–1] for WC, [0.62–1] for H2, [0.91–1] for H3, [0.95–1] for H4 (see Fig. 4). At the level of individual patients, sensitivity was in the range [0.92–0.95] for patient 1, [0.8–1] for patient 2, [0.62–0.99] for patient 3 and 1 for patient 4 and 5 (see Fig. 4).

**Fig. 5:**
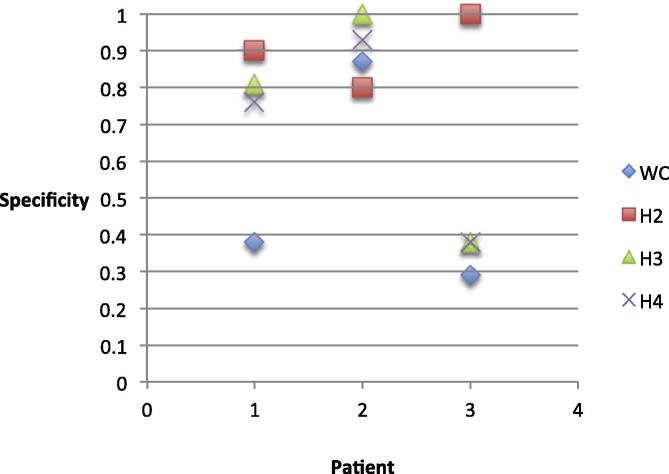
Specificity of IED marking of WC and H2, H3 and H4 for patient 1, 2 and 4.

Across the group, spike detection specificity was in the range [0.29–0.87] for WC, [0.8–1] for H2, [0.38–1] for H3 and [0.38–0.93] for H4 (see Fig. 5). At the level of individual patients, specificity across classifiers was in the range [0.38–0.9] for patient 1, [0.8–1] for patient 2 and [0.29–1] for patient 3. There were no specificity values for patient 3 and 5 due to none of the events being identified as a non-IED. Of note, for patient 1, the specificity of WC was 0.38 vs 0.9 for H2, which is the largest discrepancy (see Fig. 5).

In summary, WC sensitivity is high and similar to that of the Human reviewers while its specificity is similar to that of Human reviewers for 2/3 patients.

##### 3.2.2.2 Visual comparison of IED classes and classification overlap: Case reports

In all patients, visual inspection of the class representative IEDs allowed us to find meaningful correspondences between the majority of WC and H classes. This was reflected in the classification overlap values (see Supplementary Tables 1–6 for summary). The results for two patients (patients # 2 and 3) are summarised below. Patient # 2 was chosen to illustrate WC’s capacity to identify an IED class not previously identified by H2 and H3. The results for Patient #3 were chosen as an illustration of good classification agreement between WC and all 3 H reviewers. The case reports for the other three patients can be found in the Supplementary Case Reports.

###### 3.2.2.2.1 Patient 2

WC identified three classes, H2 and H3 identified two and H4 identified five; all four classifiers identified a non-IED class (see Table 2). The numbers of events assigned to the non-IED class were 24 for WC, 29 for H2, 16 for H3 and 14 for H4 (see Table 4).

**Table 4:** Summary of the classes and the channels for each class assigned by WC, H2, H3 and H4 for patient 2 and 3.

Patient	EEG classifier											
	WC			H2			H3			H4		
2	A	DA4 DA5	51	A	DA4 DA5	71	A	DA4 DA5	84	A	DA4 DA5	45
	B	GA51 DA4 DA5	25	NS		29	NS		16	B	DA4-5 GA51-52	18
	NS		24							C	DA4-5 GA43 GA51	21
										D	GA51-52	1
										NS		14

3	A	PSMA2 PSMA3	39	A	PSMA2 PSMA3	37	A	ASMA1 ASMA2 PSMA2 PSMA3	71	A	PSMA2 PSMA3	31
	B	ASMA1 ASMA2 PSMA2 PSMA3	61	B	ASMA1 ASMA2 PSMA2 PSMA3	62	B	PSMA2 PSMA3	27	B	ASMA1 ASMA2 PSMA2 PSMA3	68
				C	PC1-5	1	C	PC1-5	1	C	PC1-5	1

*WC class A*

Fifty-one IEDs were assigned to class WC_A and involved channels DA4 and DA5 which is identical to H2_A, H3_A and H4_A (see Table 4).

The visual similarity between these classes was further reflected in the classification overlap where WC_A agreed the most with H2_A (71%), H3_A (94%) and H4_A (78%) (see Supplementary Table 3).

*WC class B*

Twenty-five IEDs were assigned to class WC_B and involved channels DA4 and DA5 with the field extending to channel GA51 (see Table 4).

This class involved similar channels for H4_B and _C for reviewer H4 but did not correspond to any of the classes for reviewers H2 and H3.

The visual similarity between WC_B and H4_B and H4_C was further reflected in the classification overlap where WC_B agrees equally with H4_B (48%) and H4_C (48%) (see Supplementary Table 3).

The visual comparison and classification overlap indicated that WC classes did not correspond to H4_D.

###### 3.2.2.2.2 Patient 3

WC identified two classes, and H2, H3 and H4 identified three classes. None of the classifiers had a non-IED class (see Table 2).

*WC class A*

Thirty-nine IEDs were assigned to class WC_A and involved the channels PSMA2 PSMA3. The channels involved in this class were identical to those in classes H2_A, H3_B and H4_A (see Table 4).

This visual similarity was further reflected in the classification overlap where WC_A agreed the most with H2_A (79%), H3_B (64%) and H4_A (64%) (see Supplementary Table 4).

*WC class B*

Sixty-one IEDs were assigned to class WC_B and involved the channels ASMA1 ASMA2 PSMA2 PSMA3. The channels involved in this class were identical to H2_B, H3_A and H4_B (see Table 4).

This visual similarity was further reflected in the classification overlap where WC_B agreed the most with H2_B (90%), H3_A (95%) and H4_B (90%) (see Supplementary Table 4).

The visual comparison and classification overlap indicated that WC classes did not correspond to classes H2_C, H3_C and H4_C.

## 4 Discussion

The focus of this work was to provide a validation framework to determine whether automated classification of epileptic spikes on icEEG can produce results comparable to those obtained by expert human observers, and apply it to a modified version of the spike classification algorithm *Wave_clus.* Our approach to validation is based on answering the question: can the new (automated) classifier provide a similar outcome to humans? We answered this question in two ways: first, by determining whether *Wave_clus* classification falls within the range of human EEG reviewer variability using information theory metrics. In this regard we found comparable overlap between *Wave_Clus*-human and inter-human classification comparisons, indicating that *Wave_clus* classifications cannot be distinguished from human results. Second, we compared the human and automated IED classifications at the level of the individual events; we found that the sensitivity of *Wave_clus* was similar to that of the humans, and that there was generally good classification overlap.

There is significant interest in the quantification of epileptic spikes recorded in icEEG using automated algorithms (Dümpelmann and Elger, 1999, Bourien et al., 2005, Valenti et al., 2006, Brown et al., 2007, Barkmeier et al., 2012, Gaspard et al., 2014). However, only a few algorithms exploit the relationship between the activity across channels (Hufnagel et al., 2000, Bourien et al., 2005), which is an important step in the human ability to distinguish between different IED types (Gotman, 1999, James et al., 1999). Some algorithms cluster IEDs visible over multiple channels based on whether they occur in a similar temporal interval (Hufnagel et al., 2000, Bourien et al., 2005) but do not take the details of the waveform into account. Our spike classification algorithm is able to cluster multiple features by considering the details of the waveform across multiple channels. We also note the lack of comparison of the results of automated IED classification with human expert observers (Hufnagel et al., 2000, Bourien et al., 2005, Janca et al., 2013). In this study we validated the performance of *Wave_clus* as an automated IED classifier by comparing it to the performance of expert EEG reviewers.

### 4.1 Validating automated icEEG waveform classification algorithms

Validating an automated algorithm often requires a gold standard to which one can compare its performance. Due to the lack of a gold standard as to what constitutes an IED, the combined opinions (e.g. consensus or majority) of a group of expert EEG reviewers can be used as what may be called a silver standard (Barkmeier et al., 2012, Halford et al., 2013, Gaspard et al., 2014), allowing calculation of sensitivity and specificity. The greater complexity of the epileptiform activity recorded intra-cranially compared to scalp EEG means that validation methods used for the latter are generally inadequate, either due to their reliance on scalp topography or on the IED field’s at the lobar level (Wilson et al., 1999, Van Hese et al., 2008, Scherg et al., 2012). As we have shown, the greater complexity means that the number of classes assigned by each reviewer can vary greatly (see Table 2 and Supplementary Table 1).

As a result, we quantified agreement using a more general, information theoretical metric (Meilă, 2007) to determine overall spike classification similarity between automated and human spike classification. The theoretical advantage of this approach is its generalisability; in particular it allows the comparison of classification results for any number of classes. The indistinguishable performance of WC spike classification to H spike classification is demonstrated in the *VI* distribution overlap between WC_all and H_all that ranges between 58% and 96% (mean 78%) across the 5 datasets (see Table 3 and Fig. 3). To help better understand these results, let us examine the results for patient 3, with the lowest *VI* distribution overlap (58%), indicating the greatest difference between WC and H classification results. We found that the overwhelming majority of events were assigned in two classes by WC and the three H reviewers, that were visually very similar (see Table 4 and Supplementary Table 4 for the classification overlap statistics). Nonetheless in this patient dataset, the human raters tend to agree amongst themselves slightly more than with WC, as reflected in the lower *VI* values for the former. We argue that this observation is not very striking from browsing the results of the event classification overlap table (e.g. Supplementary Table 4), while it is evident in Fig. 3. It is important to note that while the statistics of *VI* distribution overlap are unknown (a much greater sample would be required), there will be a lower value in any given dataset, and we argue that 58% overlap, while suggestive of a degree of WC classification bias in this particular patient, represents a good level of agreement. Second, in the absence of ground truth there will always be uncertainty about the true level of performance, and therefore it may be argued that the WC result is in fact superior in some way; in effect that humans make the same mistakes. In this regard, we note that, when applied to IED recorded on scalp EEG during fMRI, WC classification resulted in fMRI maps that had in some cases, a higher of localisation concordance with the well-characterised generators (Pedreira et al., 2014).

### 4.2 WC performance in IED marking and classification

Similarly to our previous study (Pedreira et al., 2014), we focused on the clustering of IEDs that have already been detected and therefore, did not include the automatic detection step. Instead, we allowed our expert reviewers to ‘declassify’ the IED previously labelled by H1: this seemed necessary given the anticipated results and our knowledge of the way EEG raters work, and had the benefit of allowing us to quantify sensitivity and specificity. Previous studies investigating the sensitivity of automated IED detection algorithms on icEEG have demonstrated mixed results with some algorithms having a low (between 14% and 25%) (Dümpelmann and Elger, 1999, Barkmeier et al., 2012) and some having a high (between 63% and 75%) (Brown et al., 2007, Gaspard et al., 2014) sensitivity. We found the sensitivity of WC to be high (>76%) and similar to that of our group of EEG reviewers (see Fig. 4). Furthermore, our results show that WC classifies IEDs similar to H raters (see Supplementary Table 1), and it can identify additional classes that were not initially identified by H raters. For example WC was able to find one additional class (WC class B: GA51 DA4 DA5) for patient 2 that was not identified by H2 or H3 (see Fig. 2; Supplementary Table 1), which may indicate different generators. Furthermore, WC is also able to distinguish different IED types based on the amplitude (patient 1 class A, class B – Supplementary Table 1). An important finding in this investigation was that while there was a low specificity for WC and a high specificity for H2 (see Fig. 5), the classification of IEDs was very similar for patient 4. Both WC and H2 separated IEDs occurring in channel RA1 and RA2 with regards to polarity; WC class B (RA1 RA2 −ve) agreed the most with H2 class A (RA1 RA2 −ve) – 89%, and WC class E (RA1 RA2 +ve) agreed the most with H2 class B (RA1 RA2 +ve) – 100% (see Supplementary Table 5).

Although the present work has focussed on the validation of intracranial EEG, our approach could be generalised to other automated EEG algorithms since the validation analysis does not make any assumption about the particular nature or distribution of the electrodes or the exact nature of the signal.

### 4.3 Methodological considerations and future work

Our icEEG data was acquired during fMRI scanning and therefore, requires an offline correction for the MR gradient artefact (Carmichael et al., 2012, Boucousis et al., 2012). Carmichael et al. (2012) has shown that the EEG quality, once corrected for the MR gradient artefact, is comparable to icEEG recorded outside the scanner. We also note that quantitative analysis of the same data has been done meaningfully to study the relationship between haemodynamic changes and electrophysiological features (Murta et al., 2016, Murta et al., 2017).

Concerning the selection of the channels of interest, by relying on the notes of experienced clinician and technicians, this allowed us to ignore channels that did not contain information relevant for the classification, thereby circumventing the possibility that the distribution of the epileptiform events being unduly affected by non-epileptiform events. This approach also has the benefits of being independent of our judgement (as investigators), thereby possibly reducing bias, and having some clinical grounding (and therefore greater relevance). The issue of the method for the selection of the channels of interest may be addressed in the context of a study on automated IED detection.

Regarding the sample size used for our validation analysis, our preliminary finding as part of an imaging study is that the number and characteristics of the classes found by WC was the same when applied to the entire recordings. This provides additional evidence of the validity of our findings. We also note the lack of comparable study to provide us with a suitable standard. As an alternative comparison, for IED detection algorithm validation, we find sample sizes ranging from 279 to 6534 IEDs (Dümpelmann and Elger, 1999, Barkmeier et al., 2012, Gaspard et al., 2014, Janca et al., 2015) however, detection is a much less complex and arduous task than IED classification (Gotman, 1999, James et al., 1999). Furthermore, fatigue and error of the EEG reviewer can be a source of error in IED marking (Barkmeier et al., 2012) which may also result in erroneous IED classification. By keeping our IED sample size to 100 per recording (for a total sample size of 500), we minimised human rater fatigue and related error. Our human observers noted that while they found the task demanding, they felt that their performance level was sustainable throughout.

Training bias has been reported as a possible explanation regarding disagreement between EEG reviewers (Barkmeier et al., 2012). In our study reviewer H2 and H3 were trained at the same institution however, the mean inter-rater agreement across all EEG reviewer pairs was not significantly different (see Supplementary Table 7), indicating that there was little institutional bias.

We note that automated icEEG IED detection algorithms have paid little attention to IED event classification (Dümpelmann and Elger, 1999, Brown et al., 2007, Barkmeier et al., 2012, Gaspard et al., 2014). The high sensitivity of *Wave_clus* in IED marking (see Fig. 4) as demonstrated in this study suggests that it could be combined usefully with existing automated detection algorithms. As a result *Wave_clus* can further improve the sensitivity of IED marking by eliminating false positive automated IED detections and make the process of quantifying IEDs as accurate as possible.

The results obtained in this study are encouraging enough to apply WC across the whole EEG time course to the entire dataset of IEDs. As a result this should provide a more reliable and unbiased IED classification, which can be used to quantify the IEDs based on their frequency and morphology to determine their relationship to the seizure-onset zone. Since the EEG analysed was recorded during simultaneous fMRI acquisition this provides us with a unique opportunity to localise haemodynamic changes associated with epileptic spikes at a fundamental level.

## 5 Conclusion

We describe and apply a comprehensive framework for the evaluation of automated classifications of IEDs for clinical use in icEEG, based on a set of statistical tests chosen for their generalisability. We demonstrated the framework’s utility to show that an automated waveform EEG classification algorithm (*Wave_clus*) is practically indistinguishable to that of human EEG reviewers and can occasionally identify additional IED classes. These results also suggest that *Wave_Clus* used in combination with automated spike detection algorithms, has the potential to provide a more reliable identification of the irritative zone.
