# Supplementary material for: A novel scheme for the validation of an automated classification method for epileptic spikes by comparison with multiple observers
Source: Clin Neurophysiol. 2017 Jul;128(7):1246–54. doi: 10.1016/j.clinph.2017.04.016 (PMC5476904; doi:10.1016/j.clinph.2017.04.016)
Supplement: Supplementary data 1 [file mmc1.docx]

**Supplementary Case Reports**

***Patient 1***

WC identified 4 classes, H2 identified 9 classes and H3 and H4 identified 7 classes; all 4 classifiers had a non-IED class (see table 2). The numbers of events assigned to the non-IED class were 12 (WC), 25 (H2), 28 (H3) and 21 (H4) (see Supplementary Table 1).

*WC Class A*

Forty-four IEDs were assigned to class WC_A and involved channels G4 and G5 which is identical to classes H2_A and H4_A, and involved 50% of the channels in H3_A (see Supplementary Table 1). The visual similarity between these classes was further reflected in the classification overlap where WC_A agreed the most with H2_A (57%), H3_A (82%) and H4_A (53%) (see Supplementary Table 2).

*WC Class B*

Eleven IEDs were assigned to class WC_B and involved the same channels as WC_A but are distinguished by their amplitudes (|A| < |B|). Similar to WC_A, the channels involved in WC_B were identical to those for class H2_A, and involved 50% of the channels in H3_A (see Supplementary Table 1). The visual similarity between classes was further reflected in the classification overlap where WC_B agreed the most with H2_A (55%) and H3_A (82%). Although WC_B involved the same channels as H4_A, WC_B only had the second highest agreement with H4_A (36%) (see Supplementary Table 2).

*WC Class C*

Thirty-three IEDs were assigned to class WC_C and involved channels G13 G21 G22 DP2 and DP3 which is identical to H4_C (see Supplementary Table 1). The visual similarity between these classes was further reflected in the classification overlap and WC_C agreed the most with H4_C (39%) (see Supplementary Table 2).

Visual comparison and classification overlap revealed that classes H2_C, _D, _E, _F, _G, _H, H3_B, _C, _D, _E, _F, H4_D, _E, _F did not correspond to any WC class.

***Patient 4***

WC identified six classes, H2 and H3 identified seven classes and H4 identified six classes; all 4 classifiers had a non-IED class (see table 2). The numbers of events assigned to a non-IED class were 24 (WC), 43 (H2), 11 (H3) and 6 (H4) (see Supplementary Table 1).

*WC Class A*

Thirty-seven IEDs were assigned to class WC_A and involved channel LAH1. The channels involved in this class were identical to those in H2_C, H3_E and involved 50% of the channels involved in H4_A (see Supplementary Table 1). This visual similarity was further illustrated in the classification overlap where class WC_A agreed the most with H3_E (100%) and H4_A (86%). Although WC_B involved the same channels as H2_C, WC_B agreed the most with the non-IED class for H2 (89%) (see Supplementary Table 5).

*WC Class B*

Eighteen IEDs were assigned to class WC_B and involved channels RA1 and RA2 with a negative polarity. The channels involved in this class were identical to H2_B, H3_A and H4_D (see Supplementary Table 1). This visual similarity was further illustrated in the classification overlap where WC_B agreed the most with H2_A (89%), H3_A (83%) and H4_D (94%) (see Supplementary Table 5).

*WC Class C*

Nine IEDs were assigned to class WC_C and involved channels LAH1 LPH1. The channels involved in this class were identical to H3_C and H4_B and one of these channels was present in H2_C (see Supplementary Table 1). This visual similarity was further illustrated in the classification overlap where WC_C agreed the most with H3_C (56%) and H4_B (67%) (see Supplementary Table 5). Although there was a visual similarity between WC_C and H2_C, WC only had the second highest agreement with H2_C (33%); the highest agreement was with the non-IED class (56%) (see Supplementary Table 5).

*WC Class D*

Eight IEDs were assigned to class WC_D and involved the channels RA1 RA2 and RH1. Two out of three channels involved in this class were present in H2_A and B, H3_A and H4_D (see Supplementary Table 1). This visual similarity was reflected in the classification overlap where WC_D agreed the most with H2_A (89%), H3_A (63%) and H4_D (100%) (see Supplementary Table 5). Although the number of channels involved in WC_D was identical to H4_E, there was no classification overlap between these classes (see Supplementary Table 5).

*WC Class E*

Four IEDs were assigned to class WC_E and involved the same channels as WC_B (RA1 RA2) but were distinguished due to the difference in polarity; WC_E had a negative polarity. Only EEG reviewer H2 did the same and this was shown in the visual similarity between WC_E and H2_B (see Supplementary Table 1). This visual similarity is reflected in the classification overlap where WC_E agreed the most with H2_B (100%) (see Supplementary Table 5).

The visual comparison and classification overlap indicated that WC classes did not correspond to the three classes H2_D, E and F, three classes H3 _B,D and F, nor H4 C.

***Patient 5***

WC identified three classes, H2 identified five classes, H3 identified four classes and H4 identified six classes. None of the classifiers had a non-IED class (see table 2).

*WC Class A*

Sixty-three IEDs were assigned to class WC_A and involved the channels GA1 GA2 GA10 GA18. The channels involved in this class were identical to H2_A, H3_A and H4_B (see Supplementary Table 1). This was reflected in the classification overlap where WC_A agreed the most with H2_A (86%), H3_A (90%) and H4_B (48%) (see Supplementary Table 6).

*WC Class B*

Sixteen IEDs were assigned to class WC_B and involved the channel SPBT4. The channel involved in this class was identical to H2_C, H3_B and H4_D (see Supplementary Table 1). This was reflected in the classification overlap where WC_C agreed the most with H2_C (94%), H3_B (82%) and H4_D (75%) (see Supplementary Table 6).

*WC Class C*

Twenty-one IEDs were assigned to class WC_C and involved the channels GA1 GA2 GA9 GA10 GA11 GA18 SPBT5 SPBT6. The channels involved in this class were identical to those for H2_B, H3_D and H4_C (see Supplementary Table 1). This was reflected in the classification overlap where WC_C agreed the most with H2_B (76%), H3_D (90%) and H4_C (86%) (see Supplementary Table 6).

The visual comparison and classification overlap indicated that WC classes did not correspond to 1) two H2 classes (D and E) 2) one H3_(C) 3) three H4 classes (A, E and F).
