# Supplementary material for: A novel scheme for the validation of an automated classification method for epileptic spikes by comparison with multiple observers
Source: Clin Neurophysiol. 2017 Jul;128(7):1246–54. doi: 10.1016/j.clinph.2017.04.016 (PMC5476904; doi:10.1016/j.clinph.2017.04.016)
Supplement: Supplementary data 2 [file mmc2.docx]

**Supplementary Methods**

1. Automated spike marker adjustment

We modified the IEDs’ temporal marking (by H1) by automatically adjusting them to the peak of the sharp wave across the channels of interest, as follows: a high pass filter (a second order Butterworth filter) set at 6Hz was used to eliminate the slow wave and the Global Field Power (*GFP*) was calculated. *GFP* is defined as the sum of the squared potential between all electrode pairs:

$GFP(t)= \sum_{i=1} {(u_{i}(t)- \bar{u}(t))}^{2}$ [1]

where $u_{i}$ = is the voltage at electrode *i*, $\bar{u}$ = voltage at the referential electrode. The modified marker was set to the maximum of the *GFP* within a window of +/- 12ms of the original marker.

The EEG was then band-pass filtered (2-70Hz) and the IEDs were segmented in 300ms epochs around the GFP maximum (100ms pre-peak to 200ms post-peak) and concatenated across the channels of interest to form meta-IEDs (Pedreira et al. 2014). WC was then used to perform automated classification on the meta-IEDs similarly to our previous work (Pedreira et al., 2014).

2.0 Determining a threshold of similarity between two classifications

Two classifications with perfect agreement have a *VI* value 0. In order to determine a threshold of similarity between two classifications, we generated random classifications for 50 artificial observers, as follows:

1. We established a possible maximum and minimum number of classes per dataset based on an initial evaluation of the clinical EEG report from the five patients involved in this study.
2. The number of classes assigned to each artificial observer was chosen using a random number from a uniform distribution within the range determined in i.
3. For each of the 100 IEDs a class was assigned randomly from a uniform distribution.
4. The variation of information for each possible pair of artificial classifiers was calculated.
5. Two classifications were considered similar if their *VI* value was more than 2 SD below the mean *VI* value obtained from this random sample.
