# Supplementary material for: A novel scheme for the validation of an automated classification method for epileptic spikes by comparison with multiple observers
Source: Clin Neurophysiol. 2017 Jul;128(7):1246–54. doi: 10.1016/j.clinph.2017.04.016 (PMC5476904; doi:10.1016/j.clinph.2017.04.016)
Supplement: Supplementary data 3 [file mmc3.docx]

| **EEG classifier**  **Patient** | **WC** | | | **H2** | | | **H3** | | | **H4** | | |
| --- | --- | --- | --- | --- | --- | --- | --- | --- | --- | --- | --- | --- |
| **1** | Class | Channels | Number | Class | Channels | Number | Class | Channels | Number | Class | Channels | Number |
|  | A | G4 G5 low amp | 44 | A | G4-5 | 33 | A | G4 G5 G12 G13 | 48 | A | G4-5 | 30 |
|  | B | G4 G5 high amp | 11 | B | G4 5 21 22 29 30 | 19 | B | G12 G13 | 9 | B | G4 G5 G12 G13 G21 G22 G29 G30 | 23 |
|  | C | G13 G21 G22 DP2 DP3 | 33 | C | G23 24 | 5 | C | G23 G24 | 1 | C | G12 G13 G21 G22 | 16 |
|  | NS |  | 12 | D | G21 22 | 8 | D | G21 G22 | 2 | D | G21 G22 G23 | 8 |
|  |  |  |  | E | G14-22 | 7 | E | G4 G5-7 G12 G13 G21 G22 G29 G30 | 7 | E | G20 | 1 |
|  |  |  |  | F | G20 | 1 | F | G13 G20 G21 G22 | 5 | F | G24 G25 G31 | 1 |
|  |  |  |  | G | GA1-2 | 1 | NS |  | 28 | NS |  | 21 |
|  |  |  |  | H | DP2-DP3 G13 G20-23 G29 34-38 46-48 | 1 |  | | |  | | |
|  |  |  |  | NS |  | 25 |  |  |  |  |  |  |
| **4** | A | LAH1 | 37 | A | RA1 RA2 (-ve) | 25 | A | RA1 RA2 | 21 | A | LAH1-2 | 35 |
|  | B | RA1 RA2 (-ve) | 18 | B | RA1 RA2 (+ve) | 10 | B | RA2 | 8 | B | LAH1 LPH1 | 14 |
|  | C | LAH1 LPH1 | 9 | C | LAH1 | 10 | C | LAH1 LPH1 | 8 | C | LPH1 | 7 |
|  | D | RA 1 RA2 RH1 | 8 | D | LPH1(-ve) | 3 | D | LPH2 | 3 | D | RA1-2 | 36 |
|  | E | RA1 RA2 (+ve) | 4 | E | LPH1(+ve) | 8 | E | LAH1 | 43 | E | RA1-2 RH1 | 2 |
|  | NS |  | 24 | F | RH1 | 1 | F | LAH2 | 6 | NS |  | 6 |
|  |  | | | NS |  | 43 | NS |  | 11 |  | | |
| **5** | A | GA1 GA2 GA10 GA18 | 63 | A | GA1 2 10 18 | 59 | A | GA1 2 10 18 | 59 | A | GA1 2 9 10 17 18 | 29 |
|  | B | SPBT4 | 16 | B | GA1-2 GA9 10 11 18 SPBT5-6 | 22 | B | SPBT4 | 13 | B | GA1 2 10 18 | 30 |
|  | C | GA1 GA2 GA9 GA10 GA11 GA18 SPBT5 SPBT6 | 21 | C | SPBT4 | 15 | C | SPBT4 SAT3 4 | 3 | C | GA1 2 9 10 11 18 SPBT5-6 | 25 |
|  |  |  |  | D | SPBT5-6 | 2 | D | GA 1 2 9 10 11 18 SPBT5-6 | 25 | D | SPBT4 | 12 |
|  |  |  |  | E | GA17-18 | 2 |  |  | | E | SPBT4 SAT3-4 DH1 DH2 | 3 |
|  |  |  |  |  | | |  |  |  | F | SPBT5-6 | 1 |

Supplementary Table 1| Summary of the classes and the channels for each class assigned by WC, H2, H3 and H4 for all patients 1, 4 and 5.

Supplementary Table 2| Classification overlap (%) between WC classes and classes assigned by H2 [A], H3 [B] and H4 [C] for patient 1. Note: Percentages highlighted in red show the maximum overlap.

[A]

| **WC/H2** | **A_G4_5** | **B_G4_5_21_22_29_30** | **C_G23_24** | **NS** | **D_G21_22** | **E_G14_22** | **F_G20** | **G_GA1_2** | **H_DP2_3_G13_G20_23**  **G29_G34_38_G46_48** |
| --- | --- | --- | --- | --- | --- | --- | --- | --- | --- |
| **A_G4_5_lowamp** | **56.82** | 25.00 | 2.27 | 13.64 | 2.27 | 0.00 | 0.00 | 0.00 | 0.00 |
| **B_G4_5_highamp** | **54.55** | 36.36 | 0.00 | 9.09 | 0.00 | 0.00 | 0.00 | 0.00 | 0.00 |
| **C_G13_G21_G22_DP2_3** | 3.03 | 12.12 | 12.12 | **30.30** | 18.18 | 18.18 | 3.03 | 0.00 | 3.03 |
| **NS** | 8.33 | 0.00 | 0.00 | **66.67** | 8.33 | 8.33 | 0.00 | 8.33 | 0.00 |

[B]

| **WC/H3** | **A_G4_5_G12_13** | **B_G12_13** | **C_G23_24** | **NS** | **D_G21_22** | **E_G4_5_6_7_G12_13_G21_22_G29_G30** | **F_G13_G20_21_22** |
| --- | --- | --- | --- | --- | --- | --- | --- |
| **A_G4_5_lowamp** | **81.82** | 2.27 | 0.00 | 11.36 | 0.00 | 4.55 | 0.00 |
| **B_G4_5_highamp** | **81.82** | 9.09 | 0.00 | 0.00 | 0.00 | 9.09 | 0.00 |
| **C_G13_G21_G22_DP2_3** | 6.06 | 18.18 | 3.03 | **42.42** | 6.06 | 9.09 | 15.15 |
| **NS** | 8.33 | 8.33 | 0.00 | **75.00** | 0.00 | 8.33 | 0.00 |

[C]

| **WC/H4** | **A_G4_5** | **B_G4_5_G12_13_G21_22_G29_30** | **C_G12_13_G21_G22** | **NS** | **D_G21_22_23** | **E_G20** | **F_G24_25_G31** |
| --- | --- | --- | --- | --- | --- | --- | --- |
| **A_G4_5_lowamp** | **52.27** | 36.36 | 2.27 | 6.82 | 2.27 | 0.00 | 0.00 |
| **B_G4_5_highamp** | 36.36 | **45.45** | 0.00 | 18.18 | 0.00 | 0.00 | 0.00 |
| **C_G13_G21_G22_DP2_3** | 3.03 | 3.03 | **39.39** | 33.33 | 15.15 | 3.03 | 3.03 |
| **NS** | 16.67 | 8.33 | 16.67 | **41.67** | 16.67 | 0.00 | 0.00 |

Supplementary Table 3| Classification overlap between WC classes and classes assigned by H2 [A], H3 [B] and H4 [C] for patient 2. Note: Percentages highlighted in red show the maximum overlap.

[A]

| **WC/H2** | **A_DA4_5** | **NS** |
| --- | --- | --- |
| **A_DA4_5** | **70.59** | 29.41 |
| **B_DA4_5_GA51** | **88.00** | 12.00 |
| **NS** | **54.17** | 45.83 |

[B]

| **WC/H3** | **A_DA4_5** | **NS** |
| --- | --- | --- |
| **A_DA4_5** | **94.12** | 5.88 |
| **B_DA4_5_GA51** | **100.00** | 0.00 |
| **NS** | 45.83 | **54.17** |

[C]

| **WC/H4** | **A_DA4_5** | **B_DA4_5_GA51_52** | **C_DA4_5_GA43_GA51** | **D_GA51_52** | **NS** |
| --- | --- | --- | --- | --- | --- |
| **A_DA4_5** | **78.43** | 7.84 | 9.80 | 0.00 | 3.92 |
| **B_DA4_5_GA51** | 4.00 | **48.00** | **48.00** | 0.00 | 0.00 |
| **NS** | 16.67 | 8.33 | 20.83 | 4.17 | **50.00** |

Supplementary Table 4| Classification overlap between WC classes and classes assigned by H2 [A], H3 [B] and H4 [C] for patient 3. Note: Percentages highlighted in red show the maximum overlap.

[A]

| **WC/H2** | **A_PSMA2_3** | **B_ASMA1_2_PSMA2_3** | **C_PC1_5** |
| --- | --- | --- | --- |
| **A_PSMA2_3** | **79.49** | 17.95 | 2.56 |
| **B_ASMA1_2_PSMA2_3** | 9.84 | **90.16** | 0.00 |

[B]

| **WC/H3** | **B_PSMA2_3** | **A_ASMA1_2_PSMA2_3** | **C_PC1_5** |
| --- | --- | --- | --- |
| **A_PSMA2_3** | **64.10** | 33.33 | 2.56 |
| **B_ASMA1_2_PSMA2_3** | 4.92 | **95.08** | 0.00 |

[C]

| **WC/H4** | **A_PSMA2_3** | **A_ASMA1_2_PSMA2_3** | **C_PC1_5** |
| --- | --- | --- | --- |
| **A_PSMA2_3** | **64.10** | 33.33 | 2.56 |
| **B_ASMA1_2_PSMA2_3** | 9.84 | **90.16** | 0.00 |

Supplementary Table 5| Classification overlap between WC classes and classes assigned by H2 [A], H3 [B] and H4 [C] for patient 4. Note: Percentages highlighted in red show the maximum overlap.

[A]

| **WC/H2** | **C_LAH1** | **A_RA1_2(-ve)** | **D_LPH1(-ve)** | **F_RH1** | **B_RA1_2(+ve)** | **E_LPH1(+ve)** | **NS** |
| --- | --- | --- | --- | --- | --- | --- | --- |
| **A_LAH1** | 8.11 | 0.00 | 0.00 | 0.00 | 0.00 | 2.70 | **89.19** |
| **B_RA1_2(-ve)** | 0.00 | **88.89** | 0.00 | 0.00 | 5.56 | 0.00 | 5.56 |
| **C_LAH1_LPH1** | 33.33 | 0.00 | 0.00 | 0.00 | 0.00 | 11.11 | **55.56** |
| **D_RA1_2_RH1** | 0.00 | **87.50** | 0.00 | 0.00 | 12.50 | 0.00 | 0.00 |
| **E_RA1_2(+ve)** | 0.00 | 0.00 | 0.00 | 0.00 | **100.00** | 0.00 | 0.00 |
| **NS** | 16.67 | 8.33 | 12.50 | 4.17 | 16.67 | **25.00** | 16.67 |

[B]

| **WC/H3** | **E_LAH1** | **A_RA1_2** | **C_LAH1_LPH1** | **F_LAH2** | **B_RA2** | **D_LPH2** | **NS** |
| --- | --- | --- | --- | --- | --- | --- | --- |
| **A_LAH1** | **100.00** | 0.00 | 0.00 | 0.00 | 0.00 | 0.00 | 0.00 |
| **B_RA1_2(-ve)** | 0.00 | **83.33** | 0.00 | 0.00 | 0.00 | 0.00 | 16.67 |
| **C_LAH1_LPH1** | 33.33 | 0.00 | **55.56** | 0.00 | 0.00 | 0.00 | 11.11 |
| **D_RA1_2_RH1** | 0.00 | **62.50** | 0.00 | 0.00 | 0.00 | 0.00 | 37.50 |
| **E_RA1_2(+ve)** | 0.00 | 0.00 | 0.00 | 0.00 | **100.00** | 0.00 | 0.00 |
| **NS** | 12.50 | 4.17 | 12.50 | **25.00** | 16.67 | 12.50 | 16.67 |

[C]

| **WC/H4** | **A_LAH1_2** | **D_RA1_2** | **B_LAH1_LPH1** | **E_RA1_2_RH1** | **C_LPH1** | **NS** |
| --- | --- | --- | --- | --- | --- | --- |
| **A_LAH1** | **86.49** | 0.00 | 0.00 | 0.00 | 0.00 | 13.51 |
| **B_RA1_2(-ve)** | 0.00 | **94.44** | 0.00 | 0.00 | 0.00 | 5.56 |
| **C_LAH1_LPH1** | 0.00 | 0.00 | **66.67** | 0.00 | 33.33 | 0.00 |
| **D_RA1_2_RH1** | 0.00 | **100.00** | 0.00 | 0.00 | 0.00 | 0.00 |
| **E_RA1_2(+ve)** | 0.00 | **75.00** | 0.00 | 25.00 | 0.00 | 0.00 |
| **NS** | 12.50 | **33.33** | 33.33 | 4.17 | 16.67 | 0.00 |

Supplementary Table 6| Classification overlap between WC classes and classes assigned by H2 [A], H3 [B] and H4 [C] for patient 5. Note: Percentages highlighted in red show the maximum overlap.

[A]

| **WC/H2** | **A_GA1_GA2_GA10_GA18** | **C_SPBT4** | **B_GA1_2_GA9_GA10_GA11_GA18_SPBT5_6** | **D_SPBT5_6** | **E_GA17_18** |
| --- | --- | --- | --- | --- | --- |
| **A_GA1_GA2_GA10_GA18** | **85.71** | 0.00 | 9.52 | 1.59 | 3.17 |
| **B_SPBT4** | 0.00 | **93.75** | 0.00 | 6.25 | 0.00 |
| **C_GA1_2_GA9_GA10_GA11_GA18_SPBT5_6** | 23.81 | 0.00 | **76.19** | 0.00 | 0.00 |

[B]

| **WC/H3** | **A_GA1_GA2_GA10_GA18** | **B_SPBT4** | **D_GA1_2_GA9_GA10_GA11_GA18_SPBT5_6** | **C_SPBT4_SAT3_4** |
| --- | --- | --- | --- | --- |
| **A_GA1_GA2_GA10_GA18** | **90.48** | 0.00 | 9.52 | 0.00 |
| **B_SPBT4** | 0.00 | **81.25** | 0.00 | 18.75 |
| **C_GA1_2_GA9_GA10_GA11_GA18_SPBT5_6** | 9.52 | 0.00 | **90.48** | 0.00 |

[C]

| **WC/H4** | **B_GA1_GA2_GA10_GA18** | **D_SPBT4** | **C_GA1_2_GA9_GA10_GA11_GA18_SPBT5_6** | **A_GA1_GA2_GA9_GA10_GA17_GA18** | **E_SPBT4_SAT3_4_DH1_2** | **F_SPBT5_6** |
| --- | --- | --- | --- | --- | --- | --- |
| **A_GA1_GA2_GA10_GA18** | **47.62** | 0.00 | 11.11 | 41.27 | 0.00 | 0.00 |
| **B_SPBT4** | 0.00 | **75.00** | 0.00 | 0.00 | 18.75 | 6.25 |
| **C_GA1_2_GA9_GA10_GA11_GA18_SPBT5_6** | 0.00 | 0.00 | **85.71** | 14.29 | 0.00 | 0.00 |

Supplementary Table 7| Summary of the inter-rater agreement for all H pairs. Note: Kappa values in bold indicate a good (k>0.4) inter-rater agreement. N: number of IEDs marked by both classifiers within a pair, k: the Cohen’s Kappa value.

|  | H2-H3 | | H2-H4 | | H3-H4 | |
| --- | --- | --- | --- | --- | --- | --- |
| Patient | N | κ | N | κ | N | κ |
| 1 | 62 | **0.41** | 68 | **0.493** | 63 | 0.329 |
| 2 | 58 | 0.207 | 73 | **0.567** | 67 | **0.412** |
| 3 | 100 | **1.00** | 100 | **1.00** | 100 | **1.00** |
| 4 | 49 | -0.078 | 56 | 0.11 | 83 | -0.084 |
| 5 | 100 | **1.00** | 100 | **1.00** | 100 | **1.00** |
| Mean |  | **0.51** |  | **0.63** |  | **0.53** |
